# Lipidomic Profiling Reveals Significant Perturbations of Intracellular Lipid Homeostasis in Enterovirus-Infected Cells

**DOI:** 10.3390/ijms20235952

**Published:** 2019-11-26

**Authors:** Bingpeng Yan, Zijiao Zou, Hin Chu, Gabriella Chan, Jessica Oi-Ling Tsang, Pok-Man Lai, Shuofeng Yuan, Cyril Chik-Yan Yip, Feifei Yin, Richard Yi-Tsun Kao, Kong-Hung Sze, Susanna Kar-Pui Lau, Jasper Fuk-Woo Chan, Kwok-Yung Yuen

**Affiliations:** 1 State Key Laboratory of Emerging Infectious Diseases, The University of Hong Kong, Pokfulam, Hong Kong Special Administrative Region; ybp1205@hku.hk (B.Y.); hinchu@hku.hk (H.C.); yuansf@hku.hk (S.Y.); rytkao@hku.hk (R.Y.-T.K.); khsze@hku.hk (K.-H.S.); skplau@hku.hk (S.K.-P.L.); 2 Department of Microbiology, Li Ka Shing Faculty of Medicine, The University of Hong Kong, Pokfulam, Hong Kong Special Administrative Region; u3006001@hku.hk (Z.Z.); gchan2@hku.hk (G.C.); joltsang@connect.hku.hk (J.O.-L.T.); vangor@hku.hk (P.-M.L.); yipcyril@hku.hk (C.C.-Y.Y.); 3 Hainan Medical University-The University of Hong Kong Joint Laboratory of Tropical Infectious Diseases, Hainan Medical University, Haikou 571101, China & The University of Hong Kong, Pokfulam, Hong Kong Special Administrative Region; yinfeifeiff@163.com; 4 Department of Pathogen Biology, Hainan Medical University, Haikou, Hainan 571101, China; 5 Key Laboratory of Translational Tropical Medicine, Hainan Medical University, Haikou 571101, China; 6 Carol Yu Centre for Infection, The University of Hong Kong, Pokfulam, Hong Kong Special Administrative Region; 7 The Collaborative Innovation Center for Diagnosis and Treatment of Infectious Diseases, The University of Hong Kong, Pokfulam, Hong Kong Special Administrative Region

**Keywords:** enterovirus, fatty acid, lipidomics, UPLC-ESI-Q-TOF-MS

## Abstract

Enterovirus A71 (EV-A71) and coxsackievirus A16 (CV-A16) are the most common causes of hand, foot, and mouth disease. Severe EV-A71 and CV-A16 infections may be associated with life-threatening complications. However, the pathogenic mechanisms underlying these severe clinical and pathological features remain incompletely understood. Lipids are known to play critical roles in multiple stages of the virus replication cycle. The specific lipid profile induced upon virus infection is required for optimal virus replication. The perturbations in the host cell lipidomic profiles upon enterovirus infection have not been fully characterized. To this end, we performed ultra-high performance liquid chromatography–electrospray ionization–quadrupole–time of flight-mass spectrometry (UPLC-ESI-Q-TOF-MS)-based lipidomics to characterize the change in host lipidome upon EV-A71 and CV-A16 infections. Our results revealed that 47 lipids within 11 lipid classes were significantly perturbed after EV-A71 and CV-A16 infection. Four polyunsaturated fatty acids (PUFAs), namely, arachidonic acid (AA), docosahexaenoic acid (DHA), docosapentaenoic acid (DPA), and eicosapentaenoic acid (EPA), were consistently upregulated upon EV-A71 and CV-A16 infection. Importantly, exogenously supplying three of these four PUFAs, including AA, DHA, and EPA, in cell cultures significantly reduced EV-A71 and CV-A16 replication. Taken together, our results suggested that enteroviruses might specifically modulate the host lipid pathways for optimal virus replication. Excessive exogenous addition of lipids that disrupted this delicate homeostatic state could prevent efficient viral replication. Precise manipulation of the host lipid profile might be a potential host-targeting antiviral strategy for enterovirus infection.

## 1. Introduction

Enteroviruses are non-enveloped viruses belonging to the genus *Enterovirus* in the family *Picornaviridae* that are associated with important human and mammalian diseases. Enteroviruses have non-segmented, single-stranded, positive-sense RNA genomes that measure around 7.5 kilobases [1]. There are 15 species in the genus *Enterovirus*, namely, *Enterovirus* A to L and *Rhinovirus* A to C. Among members of the species *Enterovirus* A, enterovirus A71 (EV-A71) and coxsackievirus A16 (CV-A16) are two of the most common causes of recurrent community outbreaks of hand, foot, and mouth disease (HFMD) among children worldwide and particularly in the Asia–Pacific region. For example, in mainland China, recurrent large outbreaks of HFMD involving >120,000–600,000 patients have been reported since 1998 [2,3]. Outbreaks of EV-A71 and CV-A16 infections with severe or fatal cases have also been reported in Malaysia, Hong Kong, Japan, Singapore, Taiwan, Thailand, Vietnam, and Australia [4]. Importantly, severe EV-A71 and CV-A16 infections are associated with life-threatening complications, such as aseptic meningitis, encephalitis, myocarditis, non-cardiogenic pulmonary edema with respiratory failure, and death [1,5]. However, the pathogenic mechanisms underlying these clinical and pathological features are incompletely understood. 

Lipids are known to play crucial roles in multiple stages of the viral replication cycle. Viruses, including enteroviruses, may utilize lipids as receptors or entry co-factors for virus entry [6,7], as building blocks or regulators of the viral replication complex [8,9], as well as signaling factors to facilitate the cellular distribution of viral proteins, and the trafficking, assembly, and release of virus particles [10,11]. Interestingly, viruses require a repertoire of host lipids to complete their life cycle. Inhibitions of key lipogenesis enzymes can downregulate virus replication [12]. However, when supplied in excess, these lipids can similarly perturb efficient virus replication [13]. Together, these evidences suggest that the complete lipid landscape induced upon virus infection is required for optimal virus replication. The perturbations in the host cell lipidomic profiles upon enterovirus infection have not been fully characterized.

In this study, we first established a robust platform for lipidomic analysis of enterovirus infections. We then applied this platform to perform an unbiased analysis of the host lipidome changes induced by EV-A71 and CV-A16 in rhabdomyosarcoma (RD) cells. We found that these enteroviruses perturbed the expression of multiple lipid classes in the host cells during infection. Importantly, exogenously supplying selected fatty acids significantly inhibited viral replication. These findings provided novel insights into the role of lipids in the pathogenesis and antiviral treatment of enterovirus infection.

## 2. Results

### 2.1. Analytical Method Validation

To establish a reliable platform for lipidomic analysis, we first validated the lipid coverage and liquid chromatography-mass spectrometry (LC-MS) system stability. We applied 15 representative lipid internal standards that covered 14 lipid classes in the current study (Table S1) [14]. The retention time shift and mass accuracy of lipid standards ranged from −0.12%–4.73% and −7.7–4.13 ppm, respectively. The coefficient of variation (CV) of all internal standards in cell samples and the QC samples were lower than 18% and 15%, respectively. These results indicated that the operation repeatability was satisfactory. To evaluate the stability of the analytical batch, the CV values of all mass features in the quality control (QC) samples were calculated. A total of 98.70% and 93.32% of the features measured in the negative and positive mode, respectively, exhibited CV values of <30%, indicating that the analytical batch was stable with minimal variations. Taken together, these results showed that the analytical method was reliable and the observed differences among the test samples represented true physiological differences, rather than technical errors [15].

### 2.2. Lipidomic Profile Analysis

**Figure 1:**
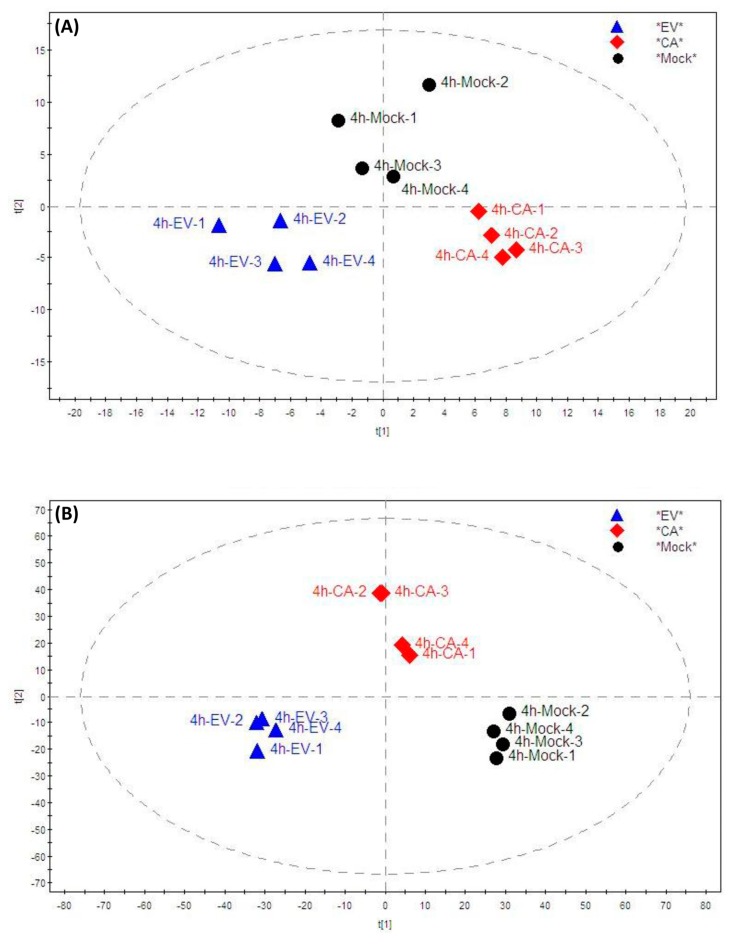
Partial least squares discriminant analysis (PLS-DA) score plots in the (**A**) negative and (**B**) positive modes showing the distribution of lipids in RD cells infected with EV-A71, CV-A16, or mock infection. Abbreviations: CA, CV-A16-infected cells; EV, EV-A71-infected cells; Mock, mock-infected cells; 4h, 4 hours post-infection.

To investigate the host cell lipid metabolism perturbations upon enterovirus infection, we compared the lipidomic profile of RD cells infected with EV-A71 or CV-A16 with that of the mock-infected cells. The lipid features in the mass spectrometry (MS) features list were first normalized with the DNA concentration of each sample. Then, the normalized list was imported into the MetaboAnalyst and SIMCA-P software for further analysis. The partial least squares discriminant analysis (PLS-DA) score plots in both the negative and positive modes were applied to visualize the distribution of lipids and discrimination between virus-infected and mock-infected samples. As shown in Figure 1, the lipid profiles of the three types of samples were clearly separated in both the negative and positive modes. This indicated that many lipid MS features exhibited obvious changes and some MS features contributed to the discrimination pattern among the three groups under the PLS-DA pattern recognition. In addition, the principal component analysis (PCA) score plots were applied to visualize the distribution of the lipids and the relatedness of virus-infected and mock-infected samples. As shown in Figure S1, all cell samples were distributed in the statistical confidence region based on the Hotelling’s T2 test. The PCA results showed that the cell samples in the current study were not outliers.

**Figure 2:**
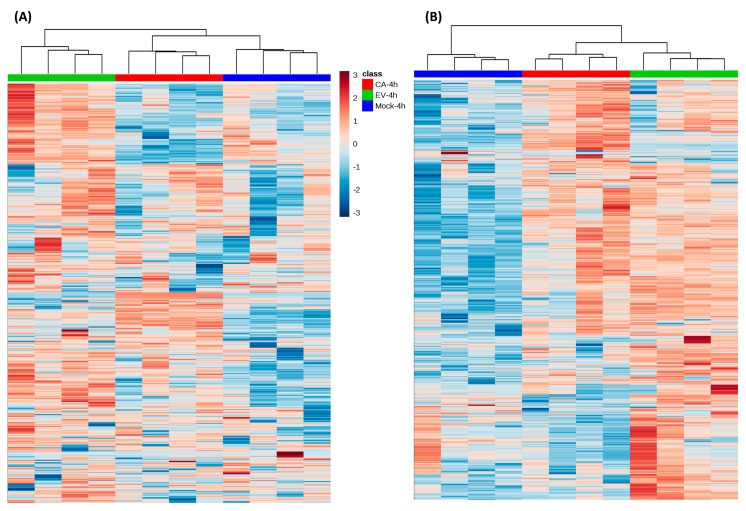
Hierarchical clustering analysis generated by MetaboAnalyst based on the all lipid features list in the (**A**) negative and (**B**) positive modes. Each bar represented a lipid feature colored in its average intensity on a normalized scale from blue (decreased) to red (increased). The dendrograms at the top were constructed based on the lipid intensity (similarity measure using Euclidean and Ward clustering algorithm). Abbreviations: CA-4h, CV-A16-infected samples harvested at 4 hours post-infection; EV-4h, EV-A71-infected samples harvested at 4 hours post-infection; Mock-4h, mock-infected samples harvested at 4 hours post-infection.

To further characterize the lipidomic changes upon EV-A71 and CV-A16 infection, a heat map was constructed based on the lipid MS features list. As shown in Figure 2, different patterns of lipidomic changes were observed in the negative mode (Figure 2A) and positive mode (Figure 2B). While the lipid profiles of the three types of samples were less different in the negative mode (Figure 2A), the lipid profiles of the EV-A71 and CV-A16-infected samples were markedly different from that of the mock-infected samples in the positive mode (Figure 2B). The common pattern of the two detection modes was that most lipid MS features in EV-A71-infected cells were in higher abundance than those of the mock-infected controls.

### 2.3. Significantly Perturbed Lipid Features in Enterovirus Infection

To select the significantly perturbed lipid features in the lipid profiles of EV-A71 or CV-A16-infected RD cells, univariate and multivariate data analyses based on the lipid MS features list were performed. In the univariate analysis, a total of 622 features (215 known + 407 unknown lipid features) were detected in the negative mode and 1496 features (413 known + 1083 unknown lipid features) were detected in the positive mode. After applying the selection criteria of an adjusted *p* < 0.05, a total of 31 significantly different features (4.98%, negative mode) and 451 features (30.15%, positive mode) were identified between the EV-A71-infected vs. mock-infected samples. A total of 58 significantly different features (9.32%, negative mode) and 273 features (18.25%, positive mode) were identified between CV-A16-infected vs. mock-infected samples. In the multivariate analysis, these cross-validated parameters were satisfactory for the OPLS-DA model (Table S2). Our model also satisfied the permutation test (100 times), which was based on the PLS-DA model when the results of the EV-A71 or CV-A16-infected samples were compared with that of the mock-infected samples (Figure S2). Overall, these results demonstrated that our validated statistical model reliably identified the significantly perturbed lipid features in our comparative lipidomic analysis between the virus-infected and mock-infected samples.

### 2.4. Lipid Changes Specific to EV-A71 and CV-A16 Infection

**Figure 3:**
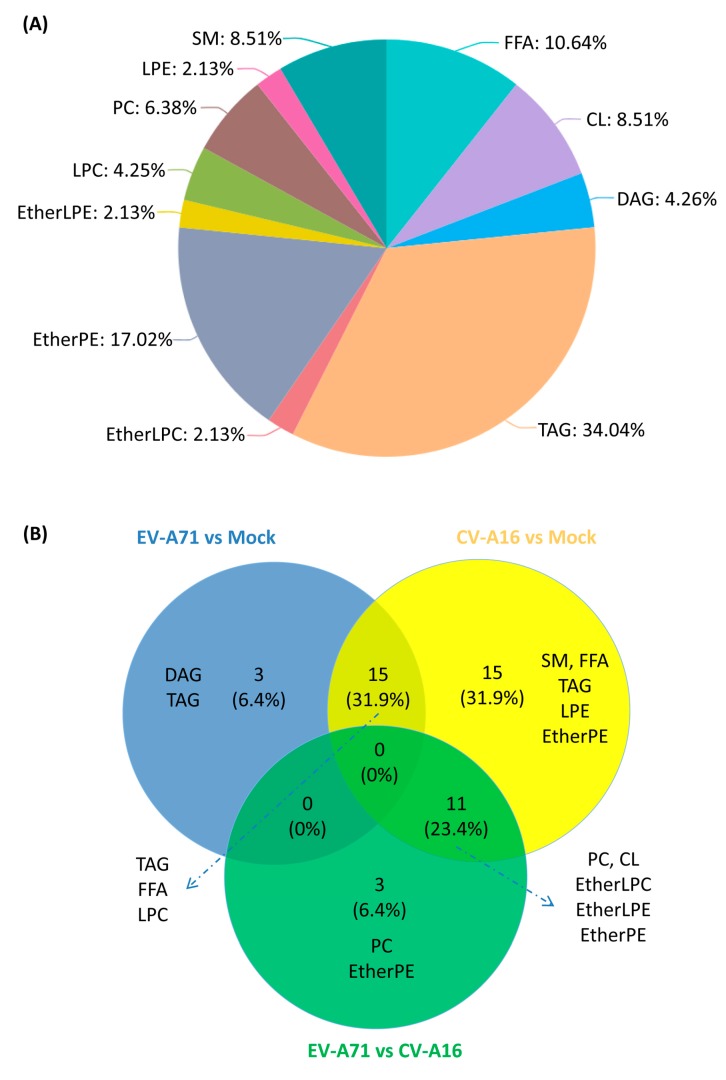
(**A**) Pie chart showing the 11 lipid classes of the 47 significantly changed lipids upon enterovirus infection. (**B**) Venn diagram demonstrating the distribution of the significantly changed lipids in the group-to-group comparison. Abbreviations: CL, cardiolipin; DAG, diacylglycerol; EtherLPC, ether-linked lysophosphatidylcholine; EtherLPE, ether-linked lysophosphatidylethanolamine; EtherPE, ether-linked phosphatidylethanolamine; FFA, free fatty acids; LPC, lysophosphatidylcholine; LPE, lysophosphatidylethanolamine; PC, phosphatidylcholine; SM, sphingomyelin; TAG, triacylglycerol.

In order to identify specific lipid changes in EV-A71 and CV-A16 infections, the significant lipid features were grouped and annotated using the MS-DIAL software [16]. Potential precursor ions were used to perform further MS/MS experiments to obtain their fragmentation patterns and the MS/MS spectra of some representative lipids were shown in Figure S3. A total of 47 significantly changed lipids were identified when the three groups of samples were compared in pairs. These lipids included those belonging to the phospholipid, glycerolipid, fatty acyls, and sphingolipid categories [17]. Detailed information of these 47 identified lipids was listed in Table 1 [18]. These 47 lipids belonged to 11 lipid classes, including cardiolipin (CL), diacylglycerol (DAG), ether-linked lysophosphatidylcholine (EtherLPC), ether-linked lysophosphatidylethanolamine (EtherLPE), ether-linked phosphatidylethanolamine (EtherPE), free fatty acids (FFA), lysophosphatidylcholine (LPC), lysophosphatidylethanolamine (LPE), phosphatidylcholine (PC), sphingomyelin (SM), and triacylglycerol (TAG) (Figure 3A) [14]. Among them, TAG (16/47, 34.0%), EtherPE (8/47, 17.0%), and FFA (5/47, 10.6%) were the three most perturbed lipid classes upon EV-A71 and CV-A16 infection. 

In the group-to-group comparison, 3/47 (6.4%) lipids belonging to diacylglycerols (DAG) and triacylglycerols (TAG) were uniquely different between EV-A71-infected vs. mock-infected cells, 15/47 (31.9%) lipids belonging to SM, FFA, TAG, LPE, and EtherPE were uniquely different between CV-A16-infected vs. mock-infected cells, and 3/47 (6.4%) lipids belonging to EtherPE and PC were uniquely different between EV-A71-infected vs. CV-A16-infected cells (Figure 3B). In addition, 15/47 (31.9%) lipids belonging to TAG, FFA, and LPC were significantly different in both EV-A71-infected and CV-A16-infected vs. mock-infected cells (Figure 3B). Taken together, these results showed that the biogenesis of diverse intracellular lipids was significantly perturbed upon EV-A71 and CV-A16 infections.

### 2.5. Characterization of the Specific Lipid Changes in EV-A71 and CV-A16 Infection

**Figure 4:**
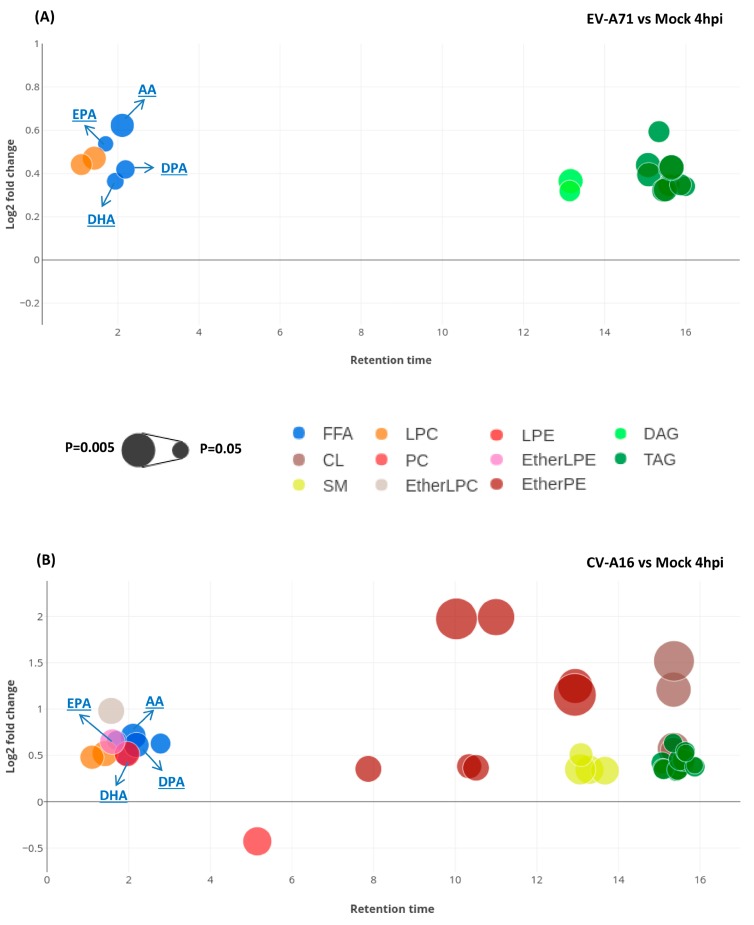
Bubble plots showing the relevant information of the significantly perturbed lipids in (**A**) EV-A71-infected vs. mock-infected cells and (**B**) CV-A16-infected vs. mock-infected cells, respectively. The X-axis is the retention time of lipids in chromatography separation. The Y-axis is the log_2_ fold change in abundance of each lipid in virus-infected cells relative to mock-infected cells. The size of each bubble represents the statistical significance of that change (*p* value by Student t-test: The larger the size, the smaller the *p* value). Abbreviations: AA, arachidonic acid; CL, cardiolipin; DAG, diacylglycerol; DHA, docosahexaenoic acid; DPA, docosapentaenoic acid; EPA, eicosapentaenoic acid; EtherLPC, ether-linked lysophosphatidylcholine; EtherLPE, ether-linked lysophosphatidylethanolamine; EtherPE, ether-linked phosphatidylethanolamine; FFA, free fatty acids; hpi, hours post-infection; LPC, lysophosphatidylcholine; LPE, lysophosphatidylethanolamine; PC, phosphatidylcholine; SM, sphingomyelin; TAG, triacylglycerol.

To further characterize the significant lipids perturbed in EV-A71 and CV-A16 infection, we next compared the direction, magnitude, and statistical significance of the EV-A71- and CV-A16-induced lipid changes in RD cells with those of the mock-infected cells at four-hours post infection (hpi) (Figure 4). EV-A71-infected cells exhibited a total of 18 significantly perturbed lipids belonging to four lipid classes, including FFA, DAG, LPC, and TAG. All of them were significantly upregulated at 4 hpi (Figure 4A and Table 1). CV-A16-infected cells exhibited a total of 41 perturbed lipids belonging to 10 lipid classes, including CL, FFA, EtherLPC, EtherLPE, EtherPE, LPC, LPE, TAG, PC, and SM. Except for PC, all of them were significantly upregulated in CV-A16 infection. Among the identified lipid classes, FFA, LPC, and TAG showed consistent upregulation in both EV-A71- and CV-A16-infected cells. These included a total of 15 lipids (4 FFAs, 2 LPCs, and 9 TAGs).

### 2.6. Antiviral Activites of the Identified Fatty Acids Against EV-A71 and CV-A16

**Figure 5:**
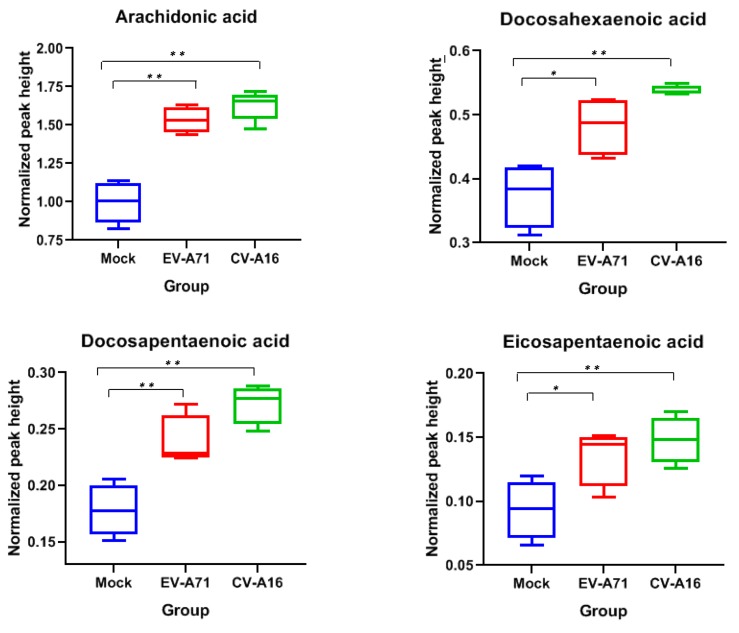
Box-whisker plots of the four fatty acid classes that were significantly upregulated in both EV-A71 and CV-A16-infected RD cells. The normalized peak heights were generated by LC-MS raw data after DNA normalization. Abbreviations: CV-A16, CV-A16-infected cells; EV-A71, EV-A71-infected cells; hpi, hours post-infection; Mock, mock-infect cells. * represented *p* < 0.05 and ** represented *p* < 0.01 (Student’s *t*-test).

**Figure 6:**
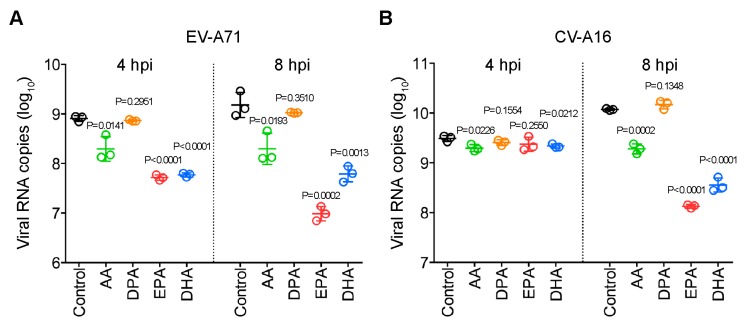
Antiviral effects of the identified fatty acids against EV-A71 (**A**) and CV-A16 (**B**). RD cells were seeded into 96-well plates to reach 90% confluency for infection and then pre-incubated with lipid-supplemented medium at the following concentrations for 2 h: AA and DPA = 100 µM, DHA = 50 µM, and EPA = 60 µM. The lipids were dissolved in 2% ethanol and 2% ethanol alone was used as “Control”. The cells were then infected with either EV-A71 or CV-A16 (MOI = 10.0). At 1 hpi, the cells were washed three times with Dulbecco’s modified eagle medium (DMEM) and then replenished with lipid-supplemented medium for 4 h of incubation. The cell lysates were then collected for qRT-PCR. Data represented means and standard deviations. Abbreviations: AA, arachidonic acid; DHA, docosahexaenoic acid; DPA, docosapentaenoic acid; EPA, eicosapentaenoic acid; hpi, hours post-infection.

Fatty acids are known to possess antiviral activities against both DNA and RNA viruses [19,20]. We therefore investigated the potential antiviral effects of the identified FFAs against EV-A71 and CV-A16. These four FFAs included arachidonic acid (AA), docosahexaenoic acid (DHA), docosapentaenoic acid (DPA), and eicosapentaenoic acid (EPA). We first confirmed the identities of AA, DHA, DPA, and EPA by matching their retention time (RT) and MS/MS fragmentation patterns with those of the authentic chemical standards. Our box-whisker plots showed that AA, DHA, DPA, and EPA indeed all exhibited significantly (*p* < 0.05) higher levels in both EV-A71- and CV-A16-infected samples at 4 hpi (Figure 5). Then, we treated EV-A71 and CV-A16-infected RD cells with AA, DHA, DPA, or EPA and assessed their anti-enteroviral effects *in vitro*. As shown in Figure 6, AA, DHA, and EPA exhibited significant (*p* < 0.001 to <0.05) antiviral effects against both EV-A71 and CV-A16 with 1–2 logs reduction in viral load.

## 3. Discussion

EV-A71 and CV-A16 are two of the most common causes of HFMD and repeatedly cause epidemics, especially among children in the Asia–Pacific region. While it is known that enteroviruses induce massive remodeling of intracellular membranes during viral replication [21,22,23], data on the cellular lipidomic profiles upon EV-A71 and CV-A16 infection are lacking. In this study, we established an ultra-high performance liquid chromatography (UPLC) MS-based lipidomics approach to characterize the host cell lipid perturbations upon enterovirus infection. Omics-based analytical method assessments and statistical analyses showed that our unbiased lipidomics approach was reliable in identifying significant differences in the host lipid profiles during enterovirus infection. We identified a total of 47 significantly changed lipids that belonged to the phospholipid, glycerolipid, fatty acyls, and sphingolipid categories among EV-A71-infected, CV-A16-infected, and mock-infected samples. These results showed that EV-A71 and CV-A16 infections significantly perturbed a wide range of host lipids.

Among the significantly changed lipids, four fatty acids, namely, AA, DHA, DPA, and EPA, were upregulated in both EV-A71 and CV-A16-infected samples, indicating their potentially important roles in *Enterovirus* A infections. In corroboration with our findings, a recent report also showed that human rhinovirus A, another important human pathogenic picornavirus, induces substantial fatty acid modifications in human bronchial epithelial cells [24]. AA, DHA, DPA, and EPA are all polyunsaturated fatty acids (PUFA). AA is an omega-6 PUFA, while DHA, DPA, and EPA are omega-3 PUFAs [25]. It has been previously shown that PUFA play key roles in inflammatory processes [9,13,26]. AA is a precursor to pro-inflammatory mediators such as prostaglandins (PG) and leukotrienes (LT), as well as lipoxins, which are endogenous anti-inflammatory, pro-resolving molecules reducing tissue injury and chronic inflammation [27]. DHA and EPA inhibit lipopolysaccharide-induced and lipopeptide-induced cyclooxygenase-2 expression and thus the inflammatory response in epithelial and dendritic cells [28,29]. Moreover, lipids derived from EPA (Resolvin E series) and DHA (Resolvin D series, maresins, and protectins) are involved in the resolution of inflammation and exhibit anti-inflammatory properties [26,30]. The omega-3 and omega-6 PUFAs interact in a delicate manner to regulate the inflammatory response. For example, omega-3 PUFAs such as DHA and EPA may serve as competitive substrates for omega-6 PUFA metabolism to decrease AA-derived eicosanoids and their effects on inflammation [31]. 

In addition to their immunomodulatory roles, the omega-3 and omega-6 PUFAs can also directly regulate virus replication. We previously demonstrated that AA and linoleic acids (LA) could inhibit the replication of coronaviruses including the Middle East respiratory syndrome coronavirus (MERS-CoV) and human coronavirus 229E (HCoV-229E) [13]. Similarly, the PUFA-derived lipid mediator protectin D1 (PD1) was reported to markedly attenuate influenza virus replication via interference with the virus RNA export machinery [26]. On the other hand, inhibition of the PUFA synthesis pathway through depleting the delta 6-desaturase enzyme or by treatment with a small-molecule inhibitor impaired hepatitis C virus (HCV) production, indicating that PUFAs could also be required for virion morphogenesis [32]. 

To investigate the biological relevance of the significantly upregulated expression of PUFAs in the context of enterovirus infection, we evaluated the effects of AA, DHA, DPA, and EPA on the replication of EV-A71 and CV-A16. We showed that three of these four PUFAs (AA, DHA, and EPA) significantly inhibited the replication of both viruses. This corroborated with our previous observation that fatty acids, including AA, possesses antiviral activity against coronaviruses [13]. Taken together, these results suggested that like coronaviruses, EV-A71 and CV-A16 might modulate specific lipid pathways in the host lipid profile to achieve an intricate homeostasis to facilitate their replication. Excessive exogenous addition of lipids might therefore disrupt this delicate homeostatic state and prevent efficient viral replication. Corroborating with our recent identification of sterol regulatory element-binding proteins (SREBPs) as a broad-spectrum antiviral treatment, precise manipulation of the host lipid profile might therefore be a potential host-targeting antiviral strategy for enterovirus infection [33]. 

## 4. Materials and Methods 

### 4.1. Viruses and Cells

RD (ATCC, CCL-136) cells were maintained in Dulbecco’s modified eagle medium (DMEM) supplemented with 10% heat-inactivated fetal bovine serum (FBS), 100U/mL penicillin, and 100g/mL streptomycin, and incubated in 5% CO_2_ at 37°C. Archived clinical isolates of EV-A71 and CV-A16 isolated from patients with HFMD were available at the Department of Microbiology, The University of Hong Kong. The viruses were cultured in RD cells in serum-free DMEM supplemented with 100 U/mL penicillin and 100 g/mL streptomycin. The supernatants were harvested when cytopathic effects (CPE) were observed and centrifuged to generate the viral stocks. The virus stocks were titrated by plaque assay on VeroE6 cells and stored at −80°C, as previously described. Briefly, confluent VeroE6 cells were infected with 10-fold serial viral dilutions. The cells were incubated with diluted viruses at 37 °C for 1 h and subsequently overlaid with 1% low-melting-point agarose (Promega, Madison, WI, USA). The cells were fixed with 4% formaldehyde as the plaques were observed and then stained with 0.2% crystal violet. All experiments involving live EV-A71 and CV-A16 followed the approved standard operating procedures of the biosafety level 2 facility at the Department of Microbiology, The University of Hong Kong, as previously described.

### 4.2. Chemical Reagents and Standards

The chemical reagents and standards for HPLC were prepared as previously described [15]. Briefly, HPLC-grade methanol, acetonitrile, chloroform, and isopropanol were purchased from Merck (Darmstadt, Germany). A Milli-Q water purification system (Millipore, Burlington, MA, USA) was used to prepare HPLC-grade water. Analytical grade formic acid, ammonium formate, and commercial standards used for biomarker identification were purchased from Sigma-Aldrich (St. Louis, MO, USA). Internal standards (IS) including Arachidonic acid-d8, Platelet-activating factor C-16-d4 (PAF C-16-d4), 22:1 Cholesterol Ester, PE (17:0/17:0), PG (17:0/17:0), PC(17:0/0:0), C17 Sphingosine, C17 Ceramide, SM (d18:1/17:0), PC (12:0/13:0), Cholesterol (d7), TG (17:0/17:1/17:0) d5, DG (12:0/12:0/0:0), DG (18:1/2:0/0:0), and PE (17:1/0:0) were purchased from Cayman Chemical (Ann Arbor, MI, USA) and Avanti Polar Lipids, Inc (Alabaster, AL, USA).

### 4.3. Lipid Treatment of EV-A71-Infected and CV-A16-Infected RD Cells

Lipid treatments of virus-infected cells were performed, as previously described, with modifications [13]. Briefly, RD cells were seeded into 96-well plates to reach 90% confluency for infection. The cells were pre-incubated with lipid-supplemented medium at the following concentrations for 2 h: AA and DPA = 100 µM, DHA = 50 µM, and EPA = 60 µM. Then, the cells were infected with either EV-A71 or CV-A16 (multiplicity of infection, MOI = 10.0). At 1 hpi, the cells were washed three times with DMEM. The cells were replenished with lipid-supplemented medium for 4 h of incubation. The cell lysates were then collected for qRT-PCR.

### 4.4. Lipid Extraction for Lipidomics Profiling

Confluent RD cells were infected with EV-A71 or CV-A16 (MOI = 10) and incubated in DMEM medium. Mock-infected RD cells were used as controls. The cells (four biological replicates per group) were collected for cellular lipid extraction at 4 hpi. Lipid extraction was performed for liquid chromatography-mass spectrometry (LC-MS) analysis according to a previously described protocol with slight modifications [34]. Briefly, 550 µL of ice-cold 150 mM ammonium bicarbonate solution was added to dissociate cells and 50 µL of cell suspension was used for DNA extraction for normalization [35]. A total of 250 µL of methanol containing IS and butylated hydroxytoluene (BHT) was added to the wells. Then, 2 mL of chloroform/methanol (v/v 2:1) was added, followed by vortexing and centrifugation at 4500 rpm for 10 min at 4 °C. The bottom phase was transferred to glass vials and dried in a Labconco Centrivap cold trap concentrator for storage at −80 ◦C. The dried samples were reconstituted in 25 µL of chloroform-methanol (1:1, v/v) and diluted to 1:10 of the original concentration of cell lysate in 225 µL IPA-ACN-water (2:1:1, v/v/v) for LC-MS analysis [36]. Inactivation of virus infectivity was confirmed before further processing, as previously described, with some modifications [13,33].

### 4.5. Ultra-High Performance Liquid Chromatography-Electrospray Ionization-Quadrupole-Time of Flight-Mass Spectrometry (UPLC-ESI-Q-TOF-MS) Analysis

UPLC-ESI-Q-TOF-MS analysis was performed, as previously described, with slight modifications [13]. Briefly, the lipid extract was analyzed using an Acquity UPLC system coupled to a Synapt G2-Si High Definition Mass Spectrometry (HDMS) system (Waters Corp., Milford, MA, USA). The chromatography was performed on a Waters ACQUITY CSH C18 column (100 × 2.1 mm; 1.7 µm) coupled to an Acquity CSH C18 VanGuard pre-column (5 × 2.1 mm; 1.7 µm) (Waters; Milford, MA, USA). The mobile phase A was 60:40 acetonitrile:water (v/v) with 10 mM ammonium formate and 0.1% formic acid. The mobile phase B for positive mode was 90:10 isopropanol:acetonitrile (v/v) with 10 mM ammonium formate and 0.1% formic acid. The mobile phase B for negative mode was 90:10 isopropanol:acetonitrile (v/v) with 10 mM ammonium acetate [37]. Gradient elution applied for UPLC-MS analysis was described in Table S3. The column and autosampler temperature were maintained at 55 °C and 4 °C, respectively. The injection volume was 7 µL. The mass spectral data were acquired in both positive and negative modes. The capillary voltage was maintained at 2.5kV (positive mode) and 2.0 kV (negative mode). The sampling cone voltage and source offset were maintained at 30 V and 60 V, respectively. Nitrogen was used as desolvation gas at a flow rate of 800 L/h. The source and desolvation temperatures were maintained at 120 °C and 400 °C, respectively. Mass spectra were acquired over the m/z range of 100–1500. The SYNAPT G2-Si HDMS system was calibrated using sodium formate clusters and operated in sensitivity mode. Leucine enkephalin was used as a lock mass for all experiments. MS/MS acquisition was operated in the same parameters as in MS acquisition. Collision energy was applied in the range of 30–45 eV for fragmentation to allow putative identification and structural elucidation of the significant lipids.

### 4.6. Analytical Method Assessment

To investigate the lipid detection and extraction capacity of the current method, a total of 15 lipid internal standards were applied for sample preparation and LC-MS analysis for monitoring the lipids coverage and extraction efficiency. Additionally, to check the stability of the LC-MS system during the analytical batch, QC samples were injected at the beginning of the run and after every six samples for monitoring the system variation. QC samples were prepared by pooling equal aliquots of each sample in the current study [38].

### 4.7. Data Processing and Statistical Analysis

Acquisition of the raw data was performed using MassLynx software version 4.1 (Waters Corp., MA, USA) and raw data were converted into the Analysis Base File (ABF) format by the Reifycs ABF Converter software (https://www.reifycs.com/AbfConverter/) [16]. The ABF data were then processed to generate a usable data matrix including annotation lipid name, accurate mass, retention time, and the corresponding intensity of unique MS features by the MS-DIAL software (http://prime.psc.riken.jp/Metabolomics_Software/MS-DIAL/) [16,39]. The data matrix was then exported as a .csv file for statistical analysis. MetaboAnalyst 3.0 (http://www.metaboanalyst.ca) and SIMCA-P V12.0 (Umetrics, Umeå, Sweden) were used for univariate and multivariate analysis, respectively [40]. For univariate analysis, the statistical significance of the features was determined by comparing the mock-infected and virus-infected groups using the Student’s t-test and the fold changes. Adjust *p*-value < 0.05, fold change >1.25 or <0.8 were used as the criteria for selecting significant features. For multivariate analysis, the features were first subjected to Pareto scaling, followed by orthogonal partial least squares discriminant analysis (OPLS-DA) to find important variables with discriminative power. Variable importance in projection (VIP) score >1 was used as a criterion for significant lipid features selection. The R2X/R2Y and Q2, which represented the X/Y variables explanation rate and the predicted ability of the OPLS-DA model, respectively, were calculated using the SIMCA-P software [41].

### 4.8. Lipids Identification

The identification of lipids was performed as previously described [13]. Briefly, MS/MS fragmentation was performed on the significant features with high abundances. The significant features were identified by searching accurate MS and MS/MS fragmentation pattern data in the MS-DIAL internal lipid database [39], MassBank of North America (MoNA, http://mona.fiehnlab.ucdavis.edu/), METLIN database (http://metlin.scripps.edu/), and LIPID MAPS (http://www.lipidmaps.org/). For confirmation of lipid identity using authentic chemical standards, the MS/MS fragmentation patterns of the chemical standards were compared with those of the candidate lipids measured under the same LC-MS condition.

## 5. Conclusions

In summary, we established and exploited a reliable unbiased lipidomic approach to characterize the host lipidomic profile changes upon EV-A71 and CV-A16 infections. Our finding of significantly upregulated expression of these PUFAs in EV-A71 and CV-A16 infections provided novel understanding into the pathogenesis and host-targeting antiviral strategies for enterovirus infections.

## Figures and Tables

**Table 1:** Lipids that were significantly different among EV-A71-infected, CV-A16-infected, and mock-infected samples.

Significant Lipids	Detection Mode	Retention Time	Accurate Mass in Detection Mode	Adduct Ion Name	Lipid Class	Identification Information	*p*-Value in EV71 Infection vs Mock	*p*-Value in CA16 Infection vs Mock	*p*-Value in CA16 vs EV71 Infection	Fold Change in EV71 Infection vs Mock	Fold Change in CA16 Infection vs Mock	Fold Change in CA16 vs EV71 Infection
Arachidonic acid	negative	2.11	303.2315	[M-H]−	FFA	STD	0.0007	0.0004	NS	1.54	1.63	1.06
Docosahexaenoic acid	negative	1.94	327.2319	[M-H]−	FFA	STD	0.0196	0.0007	NS	1.29	1.43	1.11
Docosapentaenoic acid	negative	2.19	329.2466	[M-H]−	FFA	STD	0.0095	0.0006	NS	1.34	1.53	1.14
Eicosapentaenoic acid	negative	1.7	301.2167	[M-H]−	FFA	STD	0.0382	0.0096	NS	1.45	1.58	1.09
Adrenic acid	negative	2.78	331.2624	[M-H]−	FFA	MS/MS	NS	0.0067	NS	1.25	1.55	1.23
CL (16:0/18:1/16:0/18:1)	negative	15.35	1403.989	[M-H]−	CL	MS/MS	NS	0.0000	0.0000	0.87	1.49	1.71
CL (70:5)	negative	15.35	1425.9708	[M-H]−	CL	MS/MS	NS	0.0003	0.0003	0.86	1.47	1.71
CL (16:1/18:0/18:1/18:1)	negative	15.35	1430.004	[M-H]−	CL	MS/MS	NS	0.0000	0.0000	0.91	2.31	2.54
CL (72:4)	negative	15.36	1456.0192	[M-H]−	CL	MS/MS	NS	0.0000	0.0000	0.67	2.87	4.30
SM (d14:2/28:2)	positive	13.08	809.6527	[M+H]+	SM	MS/MS	NS	0.0013	NS	1.21	1.42	1.18
SM (d18:1/24:1)	positive	13.06	813.6888	[M+H]+	SM	MS/MS	NS	0.0000	NS	1.14	1.27	1.12
SM (d18:0/24:1)	positive	13.29	815.6979	[M+H]+	SM	MS/MS	NS	0.0001	NS	1.10	1.27	1.16
SM (d18:1/24:0)	positive	13.67	815.7034	[M+H]+	SM	MS/MS	NS	0.0001	NS	1.15	1.26	1.10
PC (O-16:0/0:0)	positive	1.57	482.363	[M+H]+	EtherLPC	MS/MS	NS	0.0002	0.0007	1.23	1.97	1.60
PC (16:1/0:0)	positive	1.1	494.3254	[M+H]+	LPC	MS/MS	0.0027	0.0011	NS	1.36	1.39	1.03
PC (18:1/0:0)	positive	1.42	522.3575	[M+H]+	LPC	MS/MS	0.0008	0.0004	NS	1.39	1.43	1.04
PC (16:0/20:4)	positive	6.51	782.5679	[M+H]+	PC	MS/MS	NS	NS	0.0204	1.08	0.81	0.75
PC (20:4/20:4)	positive	5.15	830.5708	[M+H]+	PC	MS/MS	NS	0.0000	0.0000	1.12	0.74	0.66
PC (20:3/20:4)	positive	5.89	832.5861	[M+H]+	PC	MS/MS	NS	NS	0.0000	1.09	0.85	0.78
PE (P-16:0/0:0)	negative	1.6	436.2826	[M-H]−	EtherLPE	MS/MS	NS	0.0005	0.0003	1.10	1.57	1.43
PE (18:0/0:0)	negative	1.96	480.3081	[M-H]−	LPE	MS/MS	NS	0.0006	NS	1.23	1.43	1.17
PE (P-16:0/16:0)	negative	10.03	674.5099	[M-H]−	EtherPE	MS/MS	NS	0.0000	0.0000	0.98	3.92	4.02
PE (P-16:0/17:0)	negative	11	688.5256	[M-H]−	EtherPE	MS/MS	NS	0.0000	0.0000	0.92	3.99	4.33
PE (P-18:0/16:0)	negative	12.93	702.543	[M-H]−	EtherPE	MS/MS	NS	0.0000	0.0000	1.04	2.22	2.13
PE (P-16:0/16:1)	positive	7.87	674.5112	[M+H]+	EtherPE	MS/MS	NS	0.0002	NS	1.16	1.28	1.10
PE (P-16:0/18:1)	negative	10.09	700.5308	[M-H]−	EtherPE	MS/MS	NS	NS	0.0000	1.14	6.49	5.72
PE (O-16:0/18:2)	positive	10.34	702.5461	[M+H]+	EtherPE	MS/MS	NS	0.0006	NS	1.20	1.30	1.09
PE (O-18:0/16:1(9Z))	positive	12.94	704.5615	[M+H]+	EtherPE	MS/MS	NS	0.0000	0.0000	1.01	2.37	2.35
PE (P-18:0/18:2)	positive	10.51	728.5601	[M+H]+	EtherPE	MS/MS	NS	0.0003	NS	1.17	1.29	1.10
DG (18:1/18:1/0:0)	positive	13.16	638.5742	[M+NH4]+	DAG	MS/MS	0.0004	NS	NS	1.29	1.23	NS
DG (38:4)	positive	13.14	662.575	[M+NH4]+	DAG	MS/MS	0.0030	NS	NS	1.25	1.20	NS
TG (44:1)	positive	15.07	766.692	[M+NH4]+	TAG	MS/MS	0.0004	0.0033	NS	1.36	1.34	0.99
TG (46:2)	positive	15.09	792.7092	[M+NH4]+	TAG	MS/MS	0.0006	0.0069	NS	1.31	1.28	0.97
TG (14:0/16:0/16:1)	positive	15.41	794.7274	[M+NH4]+	TAG	MS/MS	NS	0.0112	NS	1.24	1.25	1.01
TG (47:1)	positive	15.57	808.7391	[M+NH4]+	TAG	MS/MS	NS	0.0023	NS	1.23	1.32	1.07
TG (14:1/16:1/18:1)	positive	15.11	818.7252	[M+NH4]+	TAG	MS/MS	NS	0.0066	NS	1.18	1.27	1.08
TG (14:0/16:1/18:1)	positive	15.43	820.7425	[M+NH4]+	TAG	MS/MS	NS	0.0064	NS	1.23	1.26	1.02
TG (49:2)	positive	15.58	834.7562	[M+NH4]+	TAG	MS/MS	0.0022	0.0026	NS	1.27	1.36	1.07
TG (16:1/16:1/18:1)	positive	15.45	846.7579	[M+NH4]+	TAG	MS/MS	0.0010	0.0043	NS	1.25	1.27	1.02
TG (16:1/17:1/18:1)	positive	15.59	860.7723	[M+NH4]+	TAG	MS/MS	NS	0.0031	NS	1.27	1.37	1.08
TG (14:0/18:1/20:4)	positive	15.34	870.757	[M+NH4]+	TAG	MS/MS	0.0025	0.0137	NS	1.51	1.55	1.03
TG (16:1/18:1/18:2)	positive	15.5	872.7713	[M+NH4]+	TAG	MS/MS	0.0008	0.0031	NS	1.25	1.37	1.10
TG (16:0/18:1/18:1)	positive	15.99	876.7005	[M+NH4]+	TAG	MS/MS	0.0062	NS	NS	1.27	1.18	NS
TG (16:1/17:1/20:1)	positive	15.87	888.8029	[M+NH4]+	TAG	MS/MS	0.0026	0.0077	NS	1.27	1.30	1.02
TG (54:5)	positive	15.64	898.7866	[M+NH4]+	TAG	MS/MS	0.0002	0.0100	NS	1.34	1.46	1.08
TG (16:0/18:1/22:5)	positive	15.65	924.8024	[M+NH4]+	TAG	MS/MS	0.0004	0.0256	NS	1.35	1.44	1.07
TG (16:0/18:1/22:4)	positive	15.87	926.8177	[M+NH4]+	TAG	MS/MS	NS	0.0326	NS	1.24	1.31	1.06

MSMS = lipids that were confirmed with fragment pattern of database; NS = not significant; STD = lipids that were confirmed with authentic standards; The ’O-’ prefix is used to indicate the presence of an alkyl ether substituent, e.g., PC (O-16:0/0:0), whereas the ’P-’ prefix is used for the 1Z-alkenyl ether (Plasmalogen) substituent, e.g., PE (P-16:0/16:0). Abbreviations: CL, cardiolipin; DAG, diacylglycerol; EtherLPC, ether-linked lysophosphatidylcholine; EtherLPE, ether-linked lysophosphatidylethanolamine; EtherPE, ether-linked phosphatidylethanolamine; FFA, free fatty acid; LPC, lysophosphatidylcholine; LPE, lysophosphatidylethanolamine; PC, phosphatidylcholine; SM, sphingomyelin; TAG, triacylglycerol.

## Supplementary Materials

Supplementary materials can be found at https://www.mdpi.com/1422-0067/20/23/5952/s1.
