# Supplementary material for: Lipidomic Profiling Reveals Significant Perturbations of Intracellular Lipid Homeostasis in Enterovirus-Infected Cells
Source: Int J Mol Sci. 2019 Nov 26;20(23):5952. doi: 10.3390/ijms20235952 (PMC6928875; doi:10.3390/ijms20235952)
Supplement: Supplementary file 1 [file ijms-20-05952-s001.pdf]

Supplementary Materials

Article

Lipidomic Profiling Reveals Significant Perturbations of Intracellular Lipid Homeostasis in Enterovirus-Infected Cells

Bingpeng Yan [1,2,†], Zijiao Zou [2,†], Hin Chu [1,2,†], Gabriella Chan [2], Jessica Oi-Ling Tsang [2], Pok-Man Lai [2], Shuofeng Yuan [1,2], Cyril Chik-Yan Yip [2], Feifei Yin [3,4,5], Richard Yi-Tsun Kao [1,2], Kong-Hung Sze [1,2], Susanna Kar-Pui Lau [1,2,7], Jasper Fuk-Woo Chan [1,2,3,7,*] and Kwok-Yung Yuen [1,2,3,7,8,*]

[1] State Key Laboratory of Emerging Infectious Diseases, The University of Hong Kong, Pokfulam, Hong Kong Special Administrative Region; ybp1205@hku.hk (B.Y.); hinchu@hku.hk (H.C.); yuansf@hku.hk (S.Y.); rytkao@hku.hk (R.Y.-T.K.); khsze@hku.hk (K.-H.S.); skplau@hku.hk (S.K.-P.L.); jfwchan@hku.hk (J.F.-W.C.); kyyuen@hku.hk (K.-Y.Y.)

[2] Department of Microbiology, Li Ka Shing Faculty of Medicine, The University of Hong Kong, Pokfulam, Hong Kong Special Administrative Region; u3006001@hku.hk (Z.Z.); gchan2@hku.hk (G.C.); joltsang@connect.hku.hk (J.O.-L.T.); vangor@hku.hk (P.-M.L.); yipcyril@hku.hk (C.C.-Y.Y.)

[3] Hainan Medical University-The University of Hong Kong Joint Laboratory of Tropical Infectious Diseases, Hainan Medical University, Haikou, Hainan, and The University of Hong Kong, Pokfulam, Hong Kong Special Administrative Region; yinfeifeiff@163.com (F.Y.)

[4] Department of Pathogen Biology, Hainan Medical University, Haikou, Hainan, 571101, China;

[5] Key Laboratory of Translational Tropical Medicine, Hainan Medical University, Haikou, Hainan, 571101, China

[7] Carol Yu Centre for Infection, The University of Hong Kong, Pokfulam, Hong Kong Special Administrative Region

[8] The Collaborative Innovation Center for Diagnosis and Treatment of Infectious Diseases, The University of Hong Kong, Pokfulam, Hong Kong Special Administrative Region

* Correspondence: jfwchan@hku.hk (J.F.-W.C.); kyyuen@hku.hk (K.-Y.Y.)
Tel.: +852-2255-2413 (J.F.-W.C. and K.-Y.Y.)
Fax: +852-2255-1241 (J.F.-W.C. and K.-Y.Y.)

† These authors contributed equally to this work as co-first authors.

Table S1. List of the mixture of 15 internal standards with corresponding statistical information

Common Name	Formula	Detection mode	Adduct ion name	m/z	RT (min)	Rt-drift	ppm tolerance	CV of cell samples	CV of QC samples
CE (22:1) [M+Na]+ iSTD	C49H86O2	Positive	[M+Na]+	729.6534	16.83	0.42%	-1.9187	14.92%	12.34%
CE (22:1) [M+NH4]+ iSTD	C49H86O2	Positive	[M+NH4]+	724.6975	16.83	0.30%	-1.2419	15.65%	14.73%
Ceramide C17 [M+H]+ iSTD	C35H69NO3	Positive	[M+H]+	552.5359	9.88	2.83%	-1.6289	14.67%	11.04%
Ceramide C17 [M+H-H2O]+ iSTD	C35H69NO3	Positive	[M+H-H2O]+	534.525	9.88	2.63%	-0.9354	9.80%	10.71%
Ceramide C17 [M+Na]+ iSTD	C35H69NO3	Positive	[M+Na]+	574.5182	9.88	2.63%	-2.0887	12.74%	11.96%
Cholesterol d7 [M-H2O+H]+ iSTD	C27H39D7O	Positive	[M-H2O+H]+	376.396	5.89	1.53%	-1.3284	16.75%	13.88%
PAF C-16 [M+H]+ d4 iSTD	C26H50D4NO7P	Positive	[M+H]+	528.3976	1.7	0.00%	-2.6495	9.13%	11.93%
DG (12:0/12:0/0:0) [M+Na]+ iSTD	C27H52O5	Positive	[M+Na]+	479.3728	4.6	1.74%	-4.3807	9.42%	10.83%
DG (12:0/12:0/0:0) [M+NH4]+ iSTD	C27H52O5	Positive	[M+NH4]+	474.4171	4.6	1.74%	-3.7941	11.69%	14.27%
DG (18:1/2:0/0:0) [M+Na]+ iSTD	C23H42O5	Positive	[M+Na]+	421.293	2.58	1.55%	-1.1868	9.94%	13.35%
DG (18:1/2:0/0:0) [M+NH4]+ iSTD	C23H42O5	Positive	[M+NH4]+	416.3379	2.58	1.55%	-1.9215	16.67%	8.87%
LPC (17:0) [M+H]+ iSTD	C25H52NO7P	Positive	[M+H]+	510.3575	1.59	2.52%	-4.1148	8.79%	11.89%
LPE (17:1) [M+H]+ iSTD	C22H44NO7P	Positive	[M+H]+	466.2947	1.29	1.55%	-4.0747	9.15%	10.26%
PC (12:0/13:0) [M+H]+ iSTD	C33H66NO8P	Positive	[M+H]+	636.4587	3.31	1.81%	1.4141	9.81%	9.45%
PE (17:0/17:0) [M+H]+ iSTD	C39H78NO8P	Positive	[M+H]+	720.5572	11.57	2.42%	-4.7186	9.51%	9.12%
SM (17:0) [M+H]+ iSTD	C40H81N2O6P	Positive	[M+H]+	717.5917	7.3	2.33%	-0.2787	8.95%	10.57%
Sphingosine (d17:1) [M+H]+ iSTD	C17H35NO2	Positive	[M+H]+	286.273	1.14	1.75%	3.8402	10.17%	10.67%
TG (17:0/17:1/17:0) [M+Na]+ d5 iSTD	C54H97D5O6	Positive	[M+H]+	874.7945	16.12	-0.12%	-7.7733	12.78%	10.08%
Arachidonic acid [M-H]- d8 iSTD	C20H24D8O2	Negative	[M-H]-	311.2837	2.04	2.39%	-1.6063	10.60%	3.09%
LPC (17:0) [M+HAc-H]- iSTD	C25H52NO7P	Negative	[M-H]-	568.3615	1.55	3.73%	-1.0557	17.30%	6.28%
LPE (17:1) [M-H]- iSTD	C22H44NO7P	Negative	[M-H]-	464.2787	1.27	3.05%	-3.6616	9.92%	6.39%
PE (17:0/17:0) [M-H]- iSTD	C39H78NO8P	Negative	[M-H]-	718.5399	11.08	4.73%	-0.6959	5.90%	7.19%
PG (17:0/17:0) [M-H] - iSTD	C40H79O10P	Negative	[M-H]-	749.5316	7.93	1.25%	4.1359	18.82%	9.00%

Abbreviations: CE, Cholesterol ester; DG, diacylglycerol; LPC, lysophosphatidylcholine; LPE, lysophosphatidylethanolamine; PC, phosphatidylcholine; PE, phosphatidylethanolamine; PG, phosphatidylglycerol; SM, sphingomyelin; TG, triacylglycerol.

Table S2. Cross-validated parameters of the OPLS-DA model based on the EV-A71-, CV-A16- and mock-infected groups

OPLS-DA model	Detection mode	Time point	R2X	R2Y	Q2
EV71 vs Mock	negative	4h	0.963	1	0.843
EV71 vs Mock	positive	4h	0.696	0.999	0.977
CA16 vs Mock	negative	4h	0.92	1	0.871
CA16 vs Mock	positive	4h	0.705	0.997	0.931

Table S3. Gradient elution program applied for UPLC-MS analysis

Time(min)	Flow rate (ml/min-1)	Mobile phase A(%)	Mobile phase B(%)	Curve
Initial	0.4	60%	40%	6
2	0.4	57%	43%	6
2.1	0.4	50%	50%	6
12	0.4	46%	54%	6
12.1	0.4	30%	70%	6
18	0.4	1%	99%	6
18.1	0.4	60%	40%	6
20	0.4	60%	40%	6

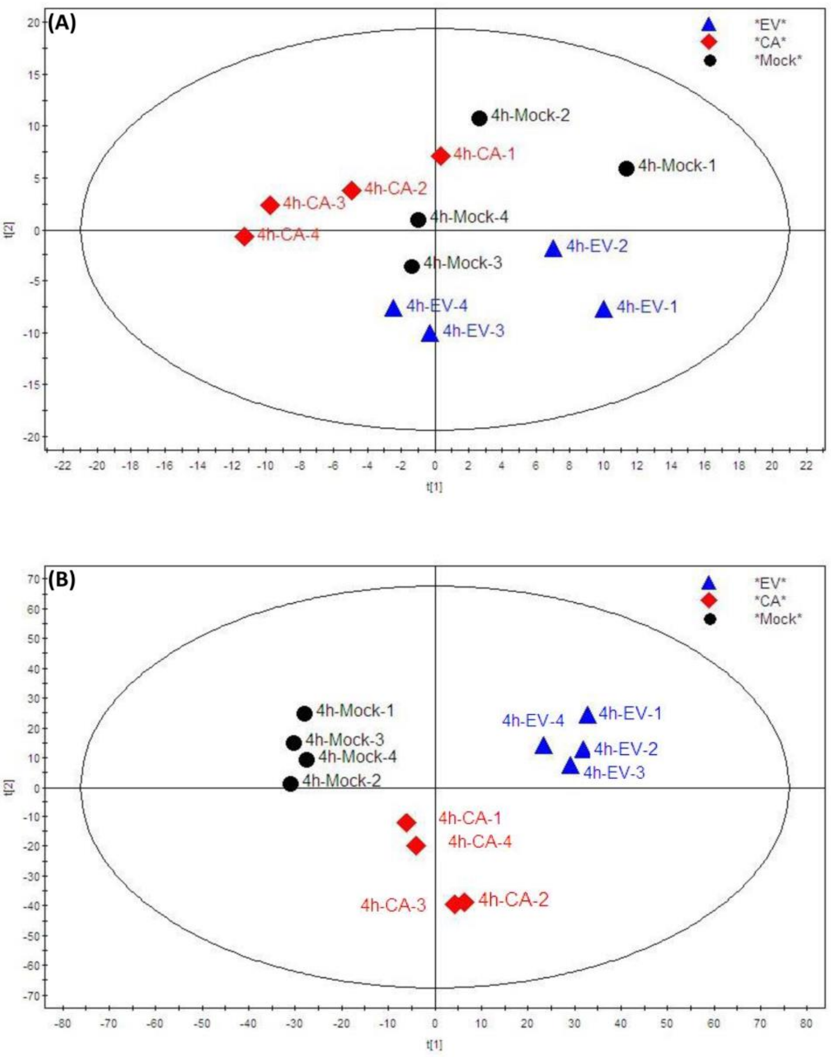

Figure S1. Principal component analysis (PCA) score plots in the (A) negative (A) and (B) positive modes (B) showing the distribution of lipids in based on RD cells samples infected with EV-A71, CV-A16, or mock infections (4 samples per group). Abbreviations: CA, CV-A16-infected cells; EV, EV-A71-infected cells; Mock, mock-infected cells; 4h, 4 hours post-infection.

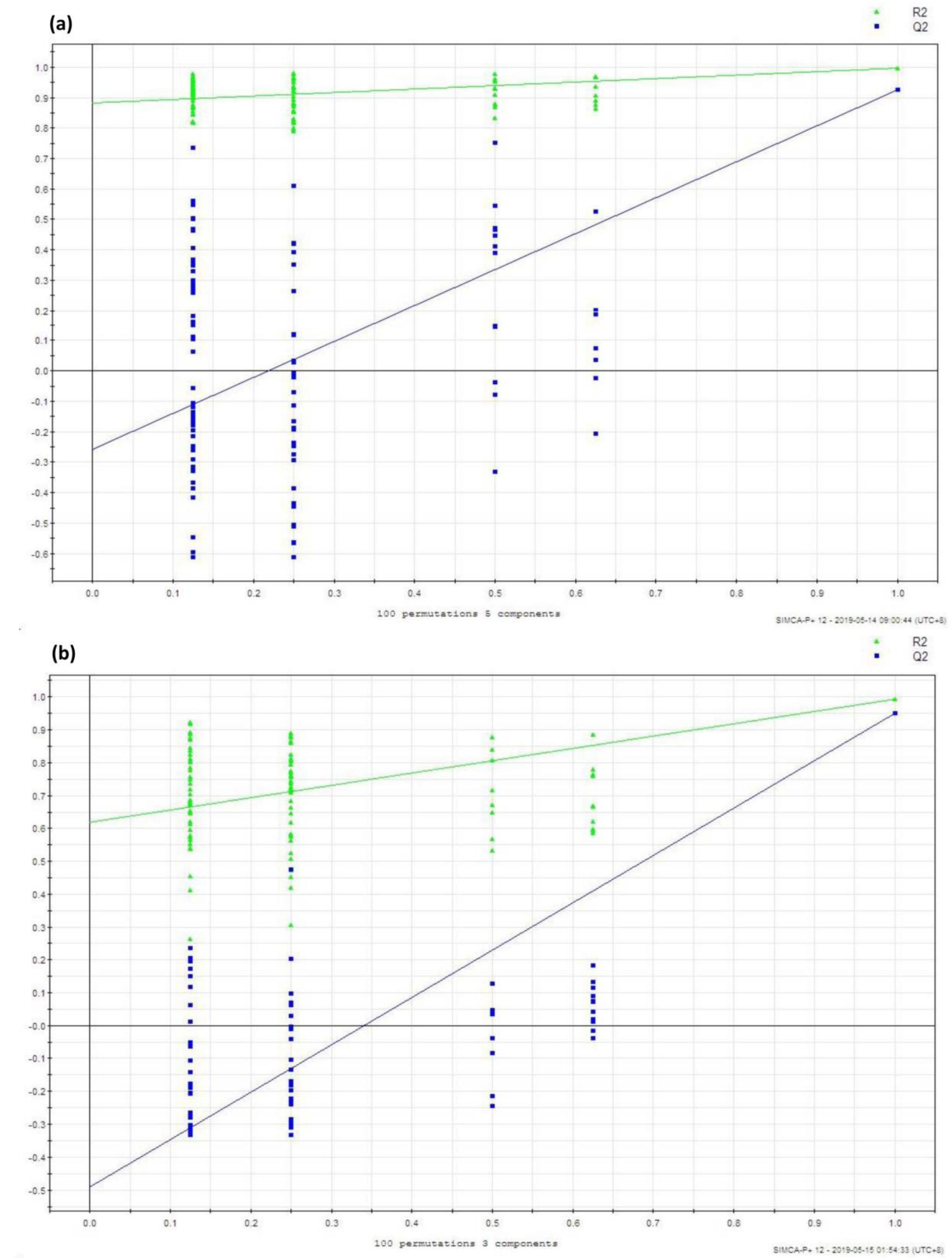

Figure S2. The permutation test (100 times) of PLS-DA model in different ionization modes. (a) Permutation test (4 hours data) in the negative mode and (b) permutation test (4 hours data) in the positive mode.

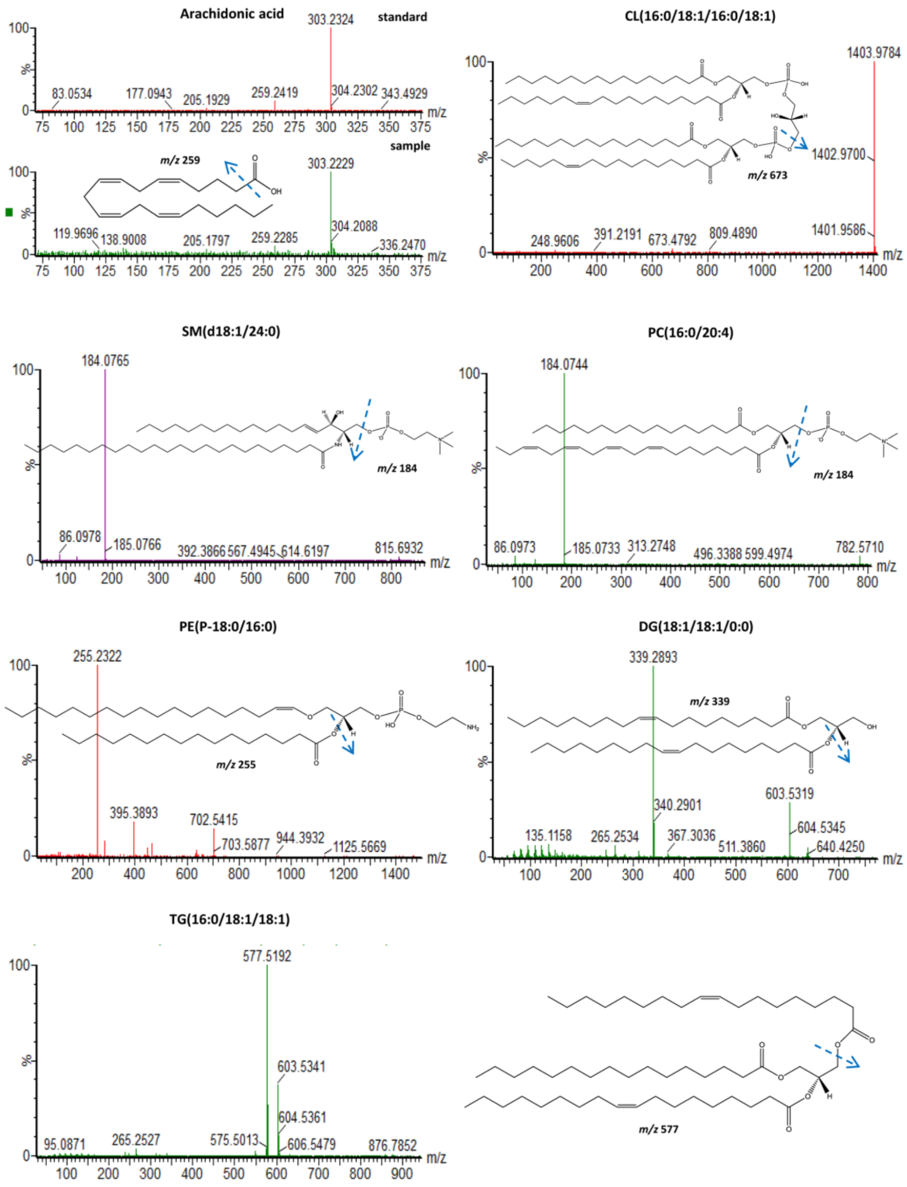

Figure S3. The MS/MS mass spectra and predicted structures with expected fragmentation profiles of the 7 representative lipids in the identified lipids table
